# Stabilised frequency of extreme positive Indian Ocean Dipole under 1.5 °C warming

**DOI:** 10.1038/s41467-018-03789-6

**Published:** 2018-04-12

**Authors:** Wenju Cai, Guojian Wang, Bolan Gan, Lixin Wu, Agus Santoso, Xiaopei Lin, Zhaohui Chen, Fan Jia, Toshio Yamagata

**Affiliations:** 1 Physical Oceanography Laboratory/CIMST, Ocean University of China and Qingdao National Laboratory for Marine Science and Technology, Yushan Road, Qingdao, 266003 China; 2 Centre for Southern Hemisphere Oceans Research (CSHOR), CSIRO Oceans and Atmosphere, Hobart, TAS 7004 Australia; 3 Australian Research Council (ARC) Centre of Excellence for Climate System Science, The University of New South Wales, Level 4 Mathews Building, Sydney, NSW 2052 Australia; 4 Institute of Oceanology, Chinese Academy of Science, Qingdao, 266071 China; 5 Application Laboratory, JAMSTEC, 3173-25 Showa-machi, Kanazawa-ku, Yokohama 236-0001 Japan

## Abstract

Extreme positive Indian Ocean Dipole (pIOD) affects weather, agriculture, ecosystems, and public health worldwide, particularly when exacerbated by an extreme El Niño. The Paris Agreement aims to limit warming below 2 °C and ideally below 1.5 °C in global mean temperature (GMT), but how extreme pIOD will respond to this target is unclear. Here we show that the frequency increases linearly as the warming proceeds, and doubles at 1.5 °C warming from the pre-industrial level (statistically significant above the 90% confidence level), underscored by a strong intermodel agreement with 11 out of 13 models producing an increase. However, in sharp contrast to a continuous increase in extreme El Niño frequency long after GMT stabilisation, the extreme pIOD frequency peaks as the GMT stabilises. The contrasting response corresponds to a 50% reduction in frequency of an extreme El Niño preceded by an extreme pIOD from that projected under a business-as-usual scenario.

## Abstract (web summary)

It is unclear how extreme positive Indian Ocean Dipole will respond to 1.5 °C of warming. Here the authors show that the frequency of these events increases linearly with warming, doubling at 1.5 °C from the pre-industrial level, but plateaus thereafter.

## Introduction

A positive Indian Ocean Dipole (pIOD) develops in boreal summer (June–August) through the Bjerknes-coupled feedback, wherein an initial cooling off the coast of Sumatra–Java suppresses local atmospheric convection, leading to anomalous easterly winds, a shoaling thermocline and stronger upwelling which in turn reinforce the initial cooling^1–3^. This positive feedback loop continues taking the pIOD to maturity in autumn (September–November). During an extreme pIOD, an additional suite of processes takes place along the equatorial Indian Ocean^3^, and the impact is far greater^1–12^, particularly when exacerbated by an ensuing extreme El Niño, as in 1997^2,13–16^. The growth of cool anomalies off Sumatra–Java induces a north-westward extension of the south-easterly trades and drying along the equatorial Indian Ocean^2^, where weak westerlies normally prevail. This additional equatorial process is a positive feedback involving anomalous zonal sea surface temperature gradient, rainfall, and zonal winds, and is strong during an extreme pIOD^3^.

During the 1997 extreme pIOD event, superimposition of these two processes generated devastating impacts. Floods and malaria outbreak in East Africa led to several thousand deaths and displaced hundreds of thousands of people^2,7,10^. In contrast, severe droughts and wildfires occurred in Indonesia^2,3,10^; the associated smoke and haze severely affected health of tens of millions of people^8,9^.

The 1997 extreme pIOD event was followed by the 1997 extreme El Niño event. While the extreme pIOD may offset some of the extreme El Niño’s impact in some places, e.g., resulting in a normal Indian monsoon^17,18^, in many other places, the impact of the 1997 extreme pIOD was exacerbated by the emerging extreme El Niño event that peaked in boreal winter, which prolonged some of the extremes and generated additional impacts^13–16,19–21^. The Intertropical Pacific Convergence Zone moved to the eastern equatorial Pacific^21^, inducing catastrophic floods in the eastern equatorial region of Ecuador and northern Peru^16^. Further, the South Pacific Convergence Zone shifted equatorward by up to 1000 km, spurring floods and droughts in south Pacific countries and shifting extreme cyclones to regions normally not affected by such events^19,20^. The sequence of an extreme pIOD preceding an extreme El Niño caused tens of billions in damage and claimed many thousands of lives worldwide^15,16^.

Under a representative concentration pathway 8.5 (RCP8.5), a business-as-usual scenario^22^ in which the global mean temperature (GMT) increases by over 4 °C by year 2100, the frequency of climate extremes, including extreme pIOD event and extreme El Niño, is projected to increase substantially^3,23^. The extent to which the aspirational target of 1.5 °C warming, or thereabout, might reduce the risk of extremes is not fully understood^24^. On the one hand, frequency of heat extremes at 1.5 °C warming relative to the pre-industrial level, though projected to increase, is 50% lower from that associated with 2 °C warming^25^; sea level rise, and the melting of the polar ice sheets, an important contributor to large-scale sea level rise, would be reduced by capping warming to 1.5 °C^26,27,28^. On the other hand, extreme El Niño frequency continues to increase for as long as a century after the GMT stabilises^29^. How the aspirational target might reduce risk of extreme pIOD is not clear.

In the Climate Model Intercomparison Project’s Phase 5 (CMIP5)^22^, the only emission pathway that may be consistent with the 1.5 °C warming target is the RCP2.6 (or RCP3PD) scenario. This emission scenario leads to low greenhouse gas concentration levels^30^ and produces a peak GMT rise close to 1.5 °C above the pre-industrial level. Only a limited number of CMIP5 models are forced with this emission scenario, possibly because such emission pathway was initially deemed less likely or too ambitious. Among them, 13 are able to generate both extreme pIOD and a 1.5 °C warming.

We analyse these 13 models, focussing on the boreal fall season (September–November) when pIOD peaks. We then calculate 31-year running averages to determine when 1.5 °C GMT warming is achieved in each model. The extreme pIOD frequency from the 31-year period centred at the 1.5 °C warming is compared with that from the last 31 years of the pre-industrial period (1869–1899), a period common across the 13 models. As such, we have a sample size of 403 years (13 models, 31 years each) for the 1.5 °C warming target and the pre-industrial period to determine the frequency change. We find that the extreme pIOD frequency increases linearly with the GMT during the transient period, and doubles at 1.5 °C warming from the pre-industrial level. The frequency increase approaches a maximum as the GMT stabilises, in sharp contrast to a continuous increase in extreme El Niño frequency long after the GMT stabilisation. In addition, the frequency of extreme pIOD event that is followed by an extreme El Niño is found to be reduced by a half from that projected under a business-as-usual scenario.

## Results

### Extreme positive IOD and the associated nonlinear processes

**Fig. 1:**
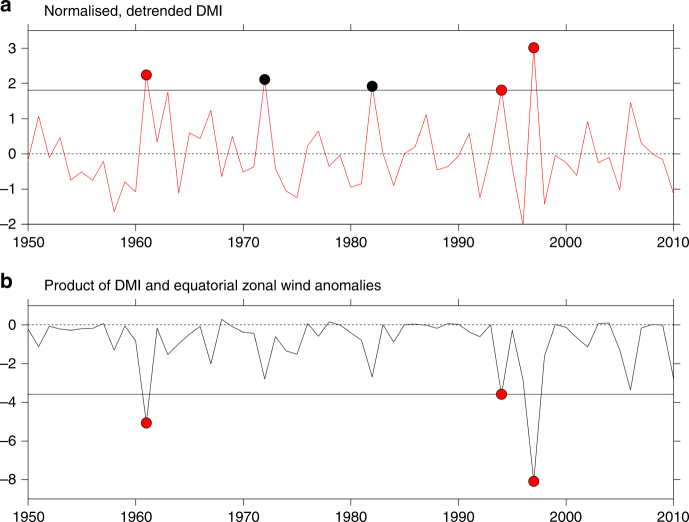
Comparison of SST-based dipole mode index and a proxy of nonlinear zonal advection. **a** The dipole mode index (DMI) as traditionally defined^1^ cannot clearly distinguish the 1961, 1994, and 1997 extreme pIOD events from other events. **b** Product of the DMI and zonal wind anomalies averaged over the equatorial Indian Ocean area (60° E–100° E, 5° S–5° N), which approximates the equatorial nonlinear zonal advection, clearly shows the distinction of the extreme pIOD from other events. Red dots indicate three observed extreme pIOD events (1961, 1994, and 1997). The black horizontal line indicates the value of the 1994 extreme pIOD, which is the weakest among the three extreme pIODs. Comparison between **a**, **b** highlights that the traditional DMI cannot distinguish extreme events from typical IOD events (indicated by black dots in **a**)

Typical pIOD events can in general be described using the dipole mode index (DMI)^1^, a measure of anomalous SST gradient across the tropical Indian Ocean (specifically western (50° E–70° E, 10° S–10° N) minus eastern (90° E–110° E, 10° S–0°) averaged SST anomalies). However, the DMI alone cannot capture extreme pIOD events which are governed by the additional suite of nonlinear processes^3^. Since 1950, there have been three extreme pIOD events, which occurred in 1961, 1994, and 1997. These extreme pIOD events differ from a typical pIOD in several respects (see ref.^3^), with an extreme pIOD featuring (1) stronger anomalous drying near Sumatra–Java, which extends westward along the equator indicating more extensive severe droughts; (2) stronger anomalous easterlies along the equator, signifying equatorial processes; and (3) stronger anomalous downstream convergence farther to the west over east Africa, leading to severe floods over the eastern African countries. This spatially complex process means that representation of extreme pIOD impacts requires more than just the commonly used DMI^1,3^, which by itself cannot tell apart the three extreme pIOD events from typical pIOD events (e.g., 1972, 1982; Fig. 1a). If the DMI alone can, then the spatial pattern between extreme and typical pIODs would be similar, but differing only in intensity. This is not the case. Thus, at least two indices are required to distinguish extreme and moderate pIOD events.

As in ref. ^3^ we used two modes of empirical orthogonal function (EOF)^31^ of rainfall anomalies averaged over September, October, and November (SON), the mature season for the IOD. The new definition is process based. The pattern of the first EOF (EOF1) of rainfall (Supplementary Fig. 1a) reflects the impact of Bjerknes feedback centred off Sumatra–Java, featuring an anomalous dry condition in the east and wet anomalies in the west, as represented by the DMI. The correlation between the observed DMI and the EOF1 time series is 0.86 over the 1979–2010 period. The pattern of EOF2 represents the impact of the additional equatorial nonlinear feedback process, i.e., nonlinear zonal advection^3^, which extends the dry anomalies from Sumatra–Java and downstream convergence westward along the equator (Supplementary Fig. 1b). A moderate pIOD is largely reflected by processes associated with EOF1. An extreme pIOD event involves the additional equatorial nonlinear feedback.

In this nonlinear zonal advection, the equatorial anomalous west-minus-east zonal SST gradient and anomalous westward-flowing oceanic zonal currents, driven by anomalous easterly winds, feed each other in a positive feedback: the anomalous winds and oceanic currents push warm water to the west, weakening the mean eastward warm water transport but deepening the thermocline to the west, driving the anomalous SST gradient, which in turn reinforces the westward winds and oceanic currents. This equatorial nonlinear oceanic zonal advection can be approximated by the product of the DMI and zonal wind anomalies along the Indian ocean equator^3^. This index, calculated using commonly used reanalysis^32,33^, successfully identifies the 1961, 1994, and 1997 extreme pIOD events (Fig. 1b), whereas the DMI cannot. We found that this nonlinear zonal advection index is significantly correlated to both EOF1 (*r* = −0.54) and EOF2 (*r* = −0.62). The implication is that EOF2 needs to be used in combination with EOF1 to identify extreme pIOD events.

Indeed, the three observed extreme pIOD events are also captured by defining an extreme pIOD as one that corresponds with EOF1 greater than 1.0 standard deviation (s.d.) and concurrent with EOF2 greater than 0.5 s.d. (see ref.^3]^). This definition, in essence, captures the physical process of an extreme pIOD; that is, strong Sumatra–Java Bjerknes feedback and the equatorial nonlinear advection feedback, while highlighting the associated extreme impacts^3^. This definition is used here to identify extreme pIODs in the CMIP5 models. The use of a s.d. (rather than an absolute value) threshold in the definition enhances intermodel comparability because the extremity of the event is measured relative to the variability magnitude within each model.

### Risk of extreme positive IOD at the 1.5 °C warming

**Fig. 2:**
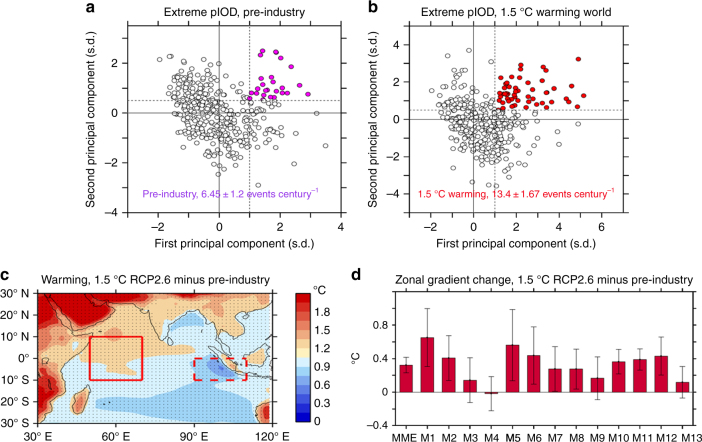
Changes associated with 1.5 °C warming from the pre-industrial level. **a**, **b** Relationship between the first and second principal component of rainfall EOF^31^ conducted over the equatorial Indian Ocean (10° S–10° N, 40° E–100° E), for the pre-industrial (1869–1899) and the targeted 1.5 °C warming levels, respectively. The results are based on 13 available models that are able to generate extreme pIOD events^3^ and can reach 1.5 °C warming under RCP2.6. The purple dots and red dots indicate extreme pIOD events for the pre-industrial and the targeted 1.5 °C warming levels, respectively. Frequency for each warming level is also indicated within each panel, with the 90% confidence intervals based on Poisson distribution. **c** Multi-model ensemble mean changes in surface temperature, created using the datasets listed in supplementary table 1. Stippled areas indicate regions where the changes are significant above the 90% confidence level, determined by a two-sided Student’s *t*-test. **d** Changes in mean zonal temperature gradient (red bars; red solid area minus red dashed area in **c**) for multi-model ensemble average and each of the 13 models (M1 to M13). The error-bars correspond to the 90% confidence level (see Methods section). Results are based on boreal fall season

At 1.5 °C warming, the extreme pIOD frequency increases from 6.5 events per century (or one event per 15 years) in the pre-industrial period (Fig. 2a, b) to 13.4 events per century (or about one event per 7 years). This is a 106% increase. The doubling in frequency is statistically significant above the 90% confidence level, according to a Poisson distribution^34^ (see Methods section). The intermodel consensus is strong, with only two out of 13 models producing a reduction (Supplementary Table 1). That is, 84% of models show an increase. There is one model (HadGEM2-AO, Supplementary Table 1) which produces a particularly large increase, from 6.5 to 29 events per century. Excluding this model, the increased frequency is still large, from 6.4 to 12.1 events per century, or 89%, statistically significant above the 90% confidence level.

The frequency increase is underpinned by the surface warming pattern, which features a faster warming in the western equatorial than the eastern equatorial Indian Ocean, leading to a robust increase in the west-minus-east zonal temperature gradient^35^ (difference between the boxed regions in Fig. 2c—the regions that are typically used to define the IOD^1^). Such a mean state is conducive to nonlinear zonal advection and hence to extreme pIOD^3,36^. This mean state change is underscored by a strong intermodel consensus (red bars in Fig. 2d). In the majority of models, the increase in the west-minus-east zonal gradient is statistically significant above the 90% confidence level (error range indicated) according to a Student’s *t*-test error estimate. The multi-model ensemble (MME) average is significant above the 99% confidence level.

Because the atmospheric convection tends to occur over the maximum SST, this mean state change makes it easier to trigger westward movement of atmosphere convection and the equatorial nonlinear zonal advection feedback^3^, the essential processes that govern extreme pIOD. This leads to a higher extreme pIOD frequency under greenhouse condition, even if temperature variability does not change^3,36^.

**Fig. 3:**
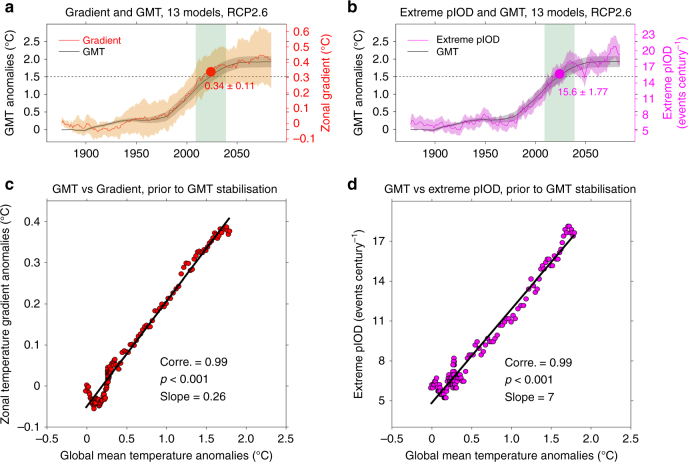
Temporal evolution of multi-model ensemble mean changes under the RCP2.6 scenario. **a** GMT anomalies (black curve) and zonal temperature gradient anomalies at the equatorial Indian Ocean (red curve) referenced to the pre-industrial condition (1869–1899) and averaged over 31-year sliding windows from 1869 to 2099 period. Their 90% confidence intervals are indicated by grey and light orange shades, respectively, based on a Student’s *t*-distribution. The value near the red circle indicates the average over the 31 years centred at 1.5 °C warming (light green-shaded zone). **b** The same as **a**, but for the extreme pIOD frequency (purple curve, events per century). The 90% confidence intervals (light purple shades) are estimated based on Poisson distribution (see Methods section). The value near the purple circle indicates the average over the 31 years centred at 1.5 °C warming (light green-shaded zone). Results are based on 13 available models. **c** Relationship between the GMT anomalies and the multi-model mean zonal temperature gradient anomalies during the period of transient increase in CO_2_. The correlation coefficient, *P*-value and slope are all indicated. **d** The same as **c**, but for the extreme pIOD frequency

The risk of extreme pIOD increases linearly with the GMT rise during the transient increase of CO_2_. This evolution is obtained by using calculation in 31-year sliding periods in each of 13 models first, and then averaging across all models (Fig. 3a, b). The MME average of west-minus-east zonal gradient (red curve in Fig. 3a) and frequency (purple curve in Fig. 3b) at 1.5 °C warming (light green-shaded region) are 0.34 °C (red filled circle, Figs. 3a), and 15.6 events per century (purple filled circle, Fig. 3b), respectively, comparable to those aggregated from outputs when each individual model is at a 1.5 °C warming (Fig. 2b, d). The results are not sensitive to the sliding periods used (Supplementary Figs. 2 and 3).

Prior to the GMT stabilisation at around 2050, the west-minus-east zonal gradient and the extreme pIOD frequency both increase approximately linearly with the GMT rise (Fig. 3c, d). This linearity is similar to the case for extreme El Niño in the pre-stabilisation period^29^, conveying a message that any rise in CO_2_ and GMT increases the risk of climate extremes. In this case, a 1.5 °C warming reduces the risk associated with a 2 °C warming by approximately 25%.

After 2050 as the GMT and greenhouse gas emissions stabilise, there is muted change in the west-minus-east zonal SST gradient that underpins an extreme pIOD (Fig. 3a), dynamically supporting a frequency stabilisation after the GMT stabilises (Fig. 3b). Although the frequency stabilisation in Fig. 3b is not well-defined over 2050–2085, with seemingly a slight MME mean trend, the MME mean trend is in fact not statistically significant. The MME mean trend of 0.10 events per century per year is far smaller than the one s.d. of the intermodel spread, which is 0.29 events per century per year. The trends in individual models show no intermodel consensus. Removing the model (CanESM2) that produces the strongest decreasing frequency trend still results in an ensemble mean trend that is not statistically significant (MME mean of 0.14 events per century per year, much smaller than the intermodel s.d. of 0.28). Removing the model (‘CSIRO-Mk3-6-0’), in which the increasing frequency trend is the largest, the MME mean frequency of the remaining 12 models shows a well-defined stabilisation (Supplementary Fig. 4). Thus, frequency of extreme pIOD reaches a maximum as the GMT stabilises.

### Contrast with projection of extreme El Niño frequency

This post-2050 evolution of the extreme pIOD is in sharp contrast to a post-stabilisation continuous increase in extreme El Niño events^29^ (see Methods section for definition of extreme El Niño). An extreme El Niño occurs when the intertropical convergence zone (ITCZ) moves to the equatorial eastern Pacific in the December, January, and February, i.e., the mature season of an El Niño, as a result of warmest SST situated in the region, or a negative off-equatorial-minus-equatorial meridional gradient. The meridional SST gradient is a barrier for movement of convection, but collapses during an extreme El Niño. The weakening of the meridional gradient continues despite the GMT stabilisation, leading to a continuous increase in extreme El Niño frequency^29^.

During the transient increase of CO_2_ (prior to 2050), the equatorial Pacific easterly winds weaken. The associated change in wind stress curls lead to a discharge of the equatorial Pacific, and ultimately a shallowing of the equatorial Pacific thermocline, analogous to the state similar to that of a positive phase of the Pacific Decadal Oscillation. The shallowing thermocline alone would be favourable to a cooling through the equatorial thermocline-SST coupling, in which a shallowing thermocline is conducive to an SST cooling, and vice versa, but the radiative forcing associated with increasing CO_2_ dominates^37,38^, leading to a fast warming in the equatorial eastern Pacific than the surrounding regions^39^.

After CO_2_ stabilisation, the equatorial Pacific easterly winds stabilise and start to strengthen; the associated wind stress curls pump heat into the ocean, and the equatorial Pacific thermocline deepens, reminiscent of a state during a negative phase of the Pacific Decadal Oscillation. The deepening thermocline is conducive to a surface warming, through the thermocline-SST coupling. This coupling is stronger in the eastern equatorial Pacific than the eastern off-equator^29^. As such, the deepening thermocline leads to a greater warming in the eastern equatorial Pacific than the off-equator, and the meridional gradient continues to weaken. Consequently, the extreme El Niño frequency continues to increase for as long as a century.

**Fig. 4:**
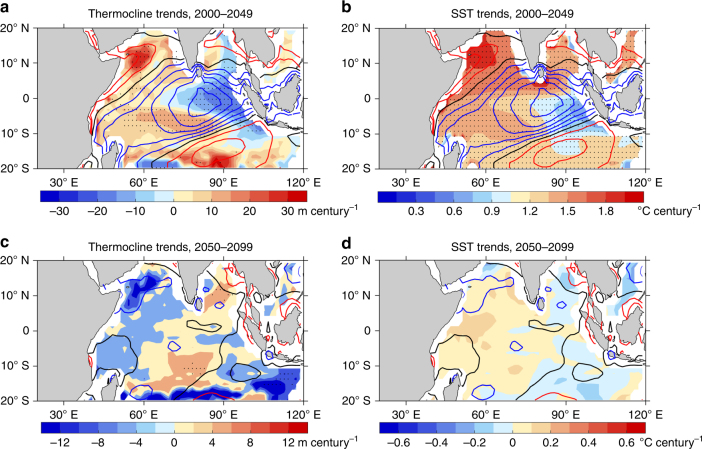
Trends in thermocline depth and SST over the tropical Indian Ocean. **a** Map of thermocline depth trends over 2000–2049, i.e., prior to the GMT stabilisation, in the unit of m per century. **b** The same as **a**, but SST (degree Celsius per century). The trends in zonal wind stress over each time period are also indicated at intervals of 0.005 Pa. Blue, red, and black contours indicate easterly, westerly, and zero zonal wind stress trends, respectively. **c**, **d** The same as **a**, **b**, but for the GMT stabilisation period (2050–2099). Stippled areas indicate statistical significance above the 95% confidence level. All panels were created using the datasets listed in supplementary table 1

In the Indian Ocean, during the transient increase of CO_2_ (prior to 2050), in association with the weakening Walker circulation, the equatorial easterly winds increase (blue contours, Fig. 4a). The enhanced easterlies induce upwelling and a shallowing thermocline in the eastern equatorial Indian Ocean (Fig. 4a), supporting a slower warming rate in the east than the west (Fig. 4b), underpinning the increased extreme pIOD frequency during the transient increase of the GMT. However, as the GMT stabilises after 2050, the weakening of the Walker circulation is halted and easterly wind trends vanish (no blue contours. Fig. 4c) leading to no further thermocline shallowing (Fig. 4c), no further cooling in the eastern equatorial Indian Ocean or no further increase in the zonal SST gradient (Fig. 4d).

The stabilisation of the zonal SST gradient underpins the result of no further increase in the extreme pIOD frequency upon the GMT stabilisation. To confirm this, we take available models (four in total) that were run beyond year 2100 (Supplementary Fig. 5; these four models are ‘CanESM2,’ ‘CESM1-CAM5,' ‘IPSL-CM5A-LR,’ and ‘MPI-ESM-LR’). Although there are still substantial interdecadal fluctuations due to the small number of models in the ensemble, the stabilisation of the zonal SST gradient and frequency is clearly shown, eventually toward a gradual reduction over the long run.

## Discussion

Our results offer incentives for the 1.5 °C warming target beyond a reduction in the extreme pIOD frequency itself from the projection under the business-as-usual emission scenario. Under RCP2.6 emission scenario, the risk of extreme pIOD frequency peaks approximately around the time of the GMT stabilisation. Amidst the continuous increase in the extreme El Niño frequency^29^, this stagnation of the extreme pIOD frequency means that the RCP2.6 post-2050 GMT stabilisation presents further benefit in that the frequency of an extreme El Niño preceded by an extreme pIOD in the previous season, as occurred in 1997, is reduced from that in the corresponding period in RCP8.5.

**Fig. 5:**
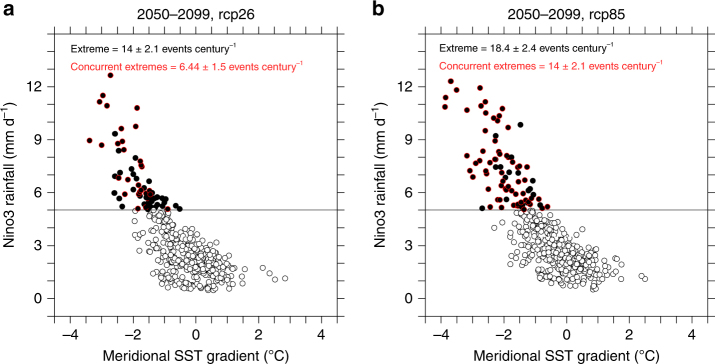
Future changes in climate extremes under different emission scenarios. The plots shown are based on nine CMIP5 models which can generate both extreme pIOD^3^ and extreme El Niño^23^ events under the RCP2.6 emission scenario, over the time period from 2050 to 2099, focusing on boreal winter (DJF) seasons when El Niño events mature. The black dots indicate extreme El Niño events; the black dot with red outline indicates an extreme El Niño preceded by an extreme pIOD event in the same year, similar to 1997. **a**, **b** Occurrences under the RCP2.6 and RCP8.5 scenario, respectively. The difference in the two emission scenarios is that the GMT stabilises after 2050 under RCP2.6, but continues to increase after 2050 under RCP8.5. The frequency (events per century) is indicated in each panel, with the 90% confidence interval based on a Poisson distribution provided

To illustrate this, we examined available models that are able to produce extreme pIOD, extreme El Niño, and a 1.5 °C warming (see Methods section) under both the RCP2.6 and the RCP8.5 scenario. A total of nine models are available. In RCP2.6, the frequency of extreme El Niño reduces by a moderate 25% from that in RCP8.5 (Fig. 5), comparing the average in the period of 2051–2099, but the frequency of an extreme El Niño preceded by an extreme pIOD is reduced by about 50% (Fig. 5; Supplementary Table 2), from 14 events per century in RCP8.5 to 6.4 events per century in RCP2.6, with only one out of nine models showing an increase. The reduction is statistically significant above the 90% confidence level. Thus, for Indonesia and other IOD-affected regions, the probability of occurrences in which the impact of an extreme pIOD is exacerbated by an extreme El Niño in the ensuing season is reduced.

Climate models suffer from biases in their simulation of the mean climate, which raises an issue as to whether our conclusions are affected in any way. We have assessed the potential impacts from the biases that are related to extreme pIOD, such as equatorial Indian Ocean zonal winds, which are in turn supported by a bias in the mean equatorial west-minus-east zonal SST gradient. For this purpose, we use an intermodel relationship between frequency change and the simulated mean climate fields, similar to the method of looking for an emergent constraint^40–42^. A statistically significant relationship suggests a potential impact if there is an associated physical process. We found no evidence of an impact on the projected extreme pIOD frequency. Similar tests of potential impact from biases in the equatorial Pacific found that if anything the Pacific cold tongue bias of SSTs, in which the eastern equatorial Pacific SSTs are too cold and extend too far west in the mean climate, may lead to an under-estimation of the projected frequency increase of extreme El Niño by introducing an overly large barrier for the ITCZ to move to the equatorial eastern Pacific. We conclude that there is no suggestion that our result is impacted by the known model biases in a simple way.

In summary, we have shown that the frequency of extreme pIOD events at 1.5 °C warming doubles that of the pre-industrial level, but the increase plateaus as the GMT stabilises, in a sharp contrast to the continued increase in the frequency of extreme El Niño long after the GMT stabilisation. During the transient increase of CO_2_, the frequency of extreme pIOD events increases linearly with the rising GMT, meaning that any increase in CO_2_ directly leads to a commensurate risk of an increased frequency of extreme pIOD. The lack of further increase in the extreme pIOD frequency after the GMT stabilises highlights a reduction in climate extremes that the aspirational warming target can bring about, and this is further underscored by a substantial reduction in the frequency of extreme El Niño events preceded by an extreme pIOD event from that projected under the business-as-usual scenario.

## Methods

### Definition of extreme positive IOD

As in ref. ^3^ two modes of EOF^31^ of rainfall anomalies are required to tell apart the impact of extreme positive IOD (pIOD) from that of moderate pIOD. The rainfall anomalies are constructed referenced to climatology over the pre-industrial level, and the EOF analysis covers the entire period.

The pattern of the first EOF (EOF1) (Supplementary Fig. 1), with an east and west dipole of reduced and enhanced convection, features particularly large anomalies of cold SST and shallow thermocline in the east, indicating Bjerknes feedback centred off Sumatra–Java. This is a common feature of all pIOD events, traditionally depicted by the DMI^1^. The pattern of EOF2, on the other hand, reflects an additional impact arising from anomalous conditions seen during extreme pIOD events, in which the warming in the equatorial west and cooling in the equatorial east strengthen the equatorial easterly anomalies, introducing an additional positive feedback along the equator, involving zonal SST gradient, zonal wind and rainfall. This along-equatorial process pushes the downstream convergence further toward the western tropical Indian Ocean and equatorial Africa, leading to the extreme floods during such events^2,11,12^. An extreme pIOD is defined as when EOF1 is greater than 1.0 s.d. and EOF2 greater than 0.5 s.d. The use of a s.d. (rather than an absolute value) threshold enhances intermodel comparability because the extremity of the event is measured relative to the variability magnitude within each model.

### Definition of extreme El Niño

As in ref. ^23^, we use a process-based rainfall definition, which facilitates a comparison of frequency with a similar impact in the pre-industrial period and the future climate. An extreme El Niño event occurs when the ITCZ, which is normally located north of the equator, moves to the equatorial eastern Pacific^20,23^ (5° S–5° N, 150° W–90° W), leading to a dramatic increase in rainfall in this normally cold and dry region. An extreme El Niño is defined as an event during which Niño3 rainfall exceeds 5 mm per day averaged over the El Niño mature season (December, January, and February).

### Eastern equatorial Pacific meridional SST gradients

Atmospheric convection occurs over regions with maximum SST, and the maximum SST is relative to surrounding regions. During extreme El Niño events, warm anomalies in the eastern equatorial Pacific weakens the meridional SST gradient, defined as the difference between the northern off-equatorial (5° N–10° N, 150° W–90° W, i.e., the present-day climatological ITCZ position) and the equatorial Pacific (2.5° S–2.5° N, 150° W–90° W). The smaller the gradient, the greater ease for this to occur.

### Model selection and analysis approach

We used CMIP5 model outputs for the boreal fall (SON) in our identification of extreme pIOD and the boreal winter (December, January, and February) in our estimate of extreme El Niño. We select a total of 13 models for their ability to simulate extreme pIOD events, and among them nine models that are able to simulate extreme El Niño as well. Models that are unable to simulate extreme pIOD in the first place clearly cannot be used to make future projections of such events. Furthermore, all the selected models must be able to reach a 1.5 °C warming under the RCP2.6 scenario. To compare the frequency at 1.5 °C warming and during the pre-industrial period, we applied a 31-year period centred at the warming of 1.5 °C, relative to the pre-industrial period of 1869–1899. The choice of the length is a balance between two factors: sufficiently long such that the number of extreme pIOD events within each window is not too small, but not too long so that warming is not either far smaller or far greater than the targeted 1.5 °C. Changing the window length to 21, 41, or 51 years does not materially alter our results (Supplementary Figs. 2–3). We used first ensemble member from each model and equal weight across all selected models in the MME average.

To assess the difference in projected change of extreme pIOD events followed by extreme El Niño events, we used common models that are forced by the RCP2.6 and RCP8.5, and are also able to produce a warming greater than 1.5 °C, extreme El Niño, and extreme pIOD. Nine models meet these criteria.

### Statistical significance tests

We used various statistical tests to assess the significance of our results. In terms of frequency of extreme pIOD and extreme El Niño events, we deployed a Poisson distribution^34^, suitable for a discrete probability distribution that expresses the probability of a given number of events occurring in a fixed interval of time. Unless otherwise stated, the 90% Poisson confidence intervals are estimated. Otherwise, we use a Student’s *t*-test, again based on the 90% confidence interval.

To gauge the uncertainties associated with the change in the zonal gradient between a 1.5 °C warming world and the pre-industrial period (Fig. 2d), in terms of a difference between two 31-year periods, for each model we first quadratically detrend the entire time series of the gradient (1869–2100), and apply a 31-year sliding window to calculate the running average of the gradient. We calculate difference between two randomly selected values 10,000 times to estimate the 90% confidence interval. In each model, if the change in the zonal gradient between a 1.5 °C warming world and the pre-industrial period is statistically significant, then the change will sit outside the appropriate range of natural variability. For the MME difference, the confidence interval is estimated based on the intermodel spread using Student’s *t*-distribution.

### Data availability

All data supporting the findings of this study are available from the corresponding authors upon request.

## Electronic supplementary material

Supplementary Information
