# Supplementary material for: Stabilised frequency of extreme positive Indian Ocean Dipole under 1.5 °C warming
Source: Nat Commun. 2018 Apr 12;9:1419. doi: 10.1038/s41467-018-03789-6 (PMC5897553; doi:10.1038/s41467-018-03789-6)
Supplement: Supplementary file 1 — Supplementary Information [file 41467_2018_3789_MOESM1_ESM.docx]

**SUPPLEMENTARY INFORMATION**

**Stabilised frequency of extreme positive Indian Ocean Dipole under 1.5 °C warming target**

Cai *et al.*


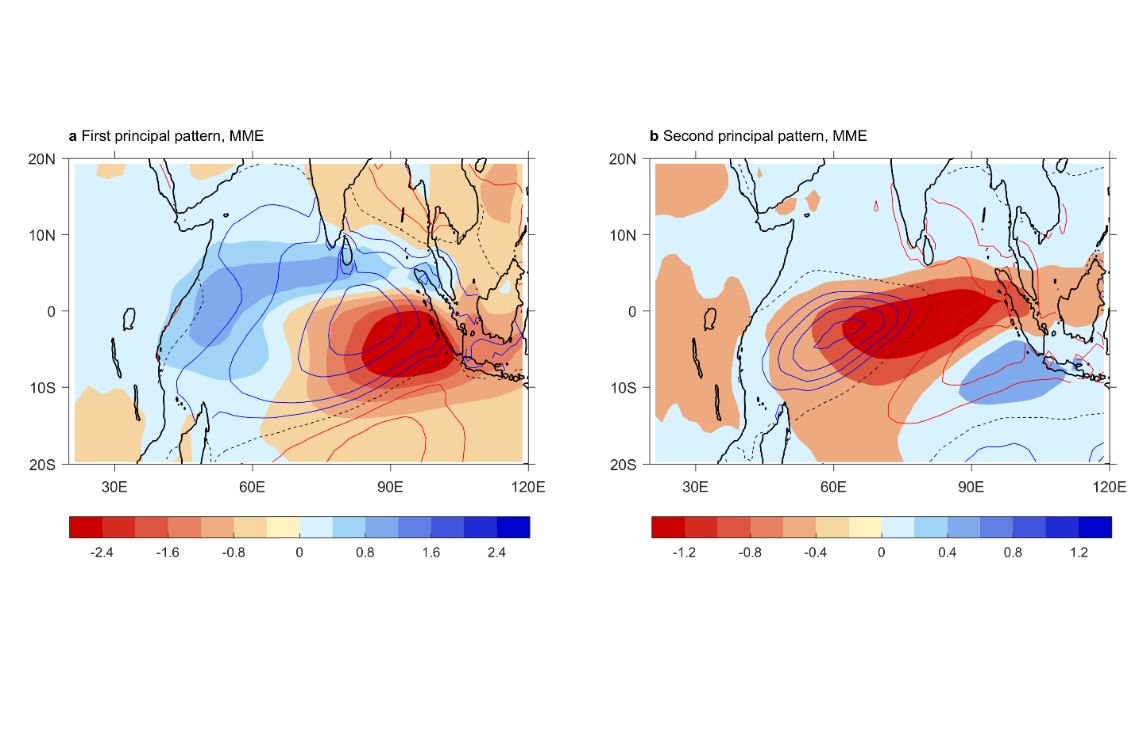


**Supplementary Figure 1 | Multi-model ensemble mean of the principal rainfall patterns**. The first (**a**) and second (**b**) principal rainfall patterns (color shading) evaluated by applying EOFs onto rainfall anomalies referenced to the rainfall climatology over the pre-industrial period (see Methods). Blue contours, red contours and black dashed contours indicate the associated easterly wind anomalies, westerly wind anomalies and zero zonal wind anomalies respectively, through regression of similarly constructed wind anomalies onto the associated time series. The results were generated using the datasets listed in supplementary table 1.


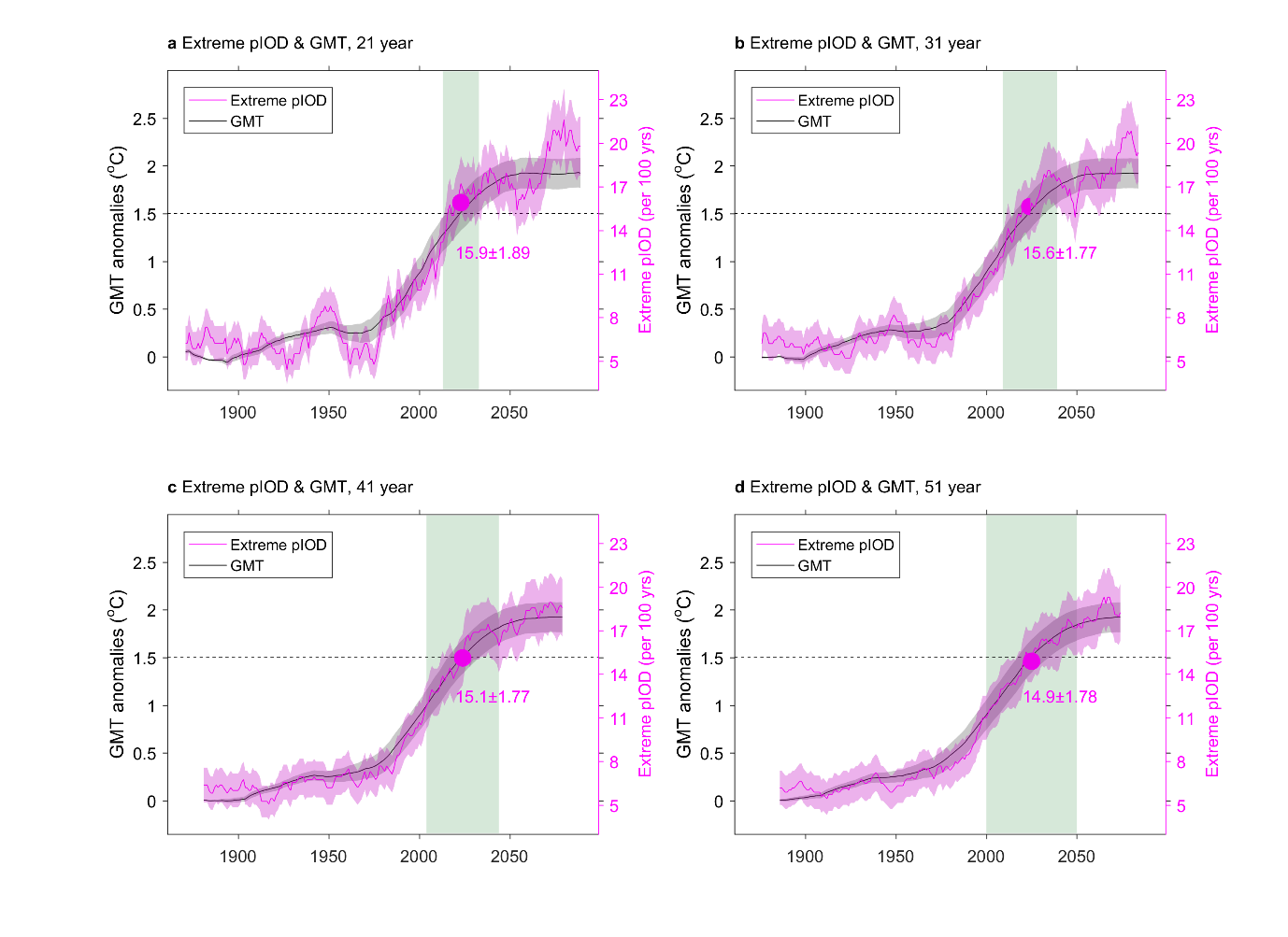


**Supplementary Figure 2 | Sensitivity of the frequency of extreme pIOD events to lengths of sliding windows.**  Evolution of multi-model ensemble averaged GMT anomalies (black curve), and the frequency of extreme pIOD events (purple curve, events per 100 years), using 13 available models under the historical and RCP2.6 emission scenario. Shown are calculated using, **a-d,** 21-, 31-, 41-, and 51-year sliding windows for each model, respectively. The 90% confidence intervals for extreme pIOD (light purple shades) and for GMT anomalies (grey shades) are based on Poisson distribution (see Methods) and Student’s *t-*distribution, respectively. The text label near the purple filled circle indicates the averaged frequency of extreme pIOD events centred at the 1.5 ^o^C warming (light shaded time window). The results show a very small difference using various window lengths.


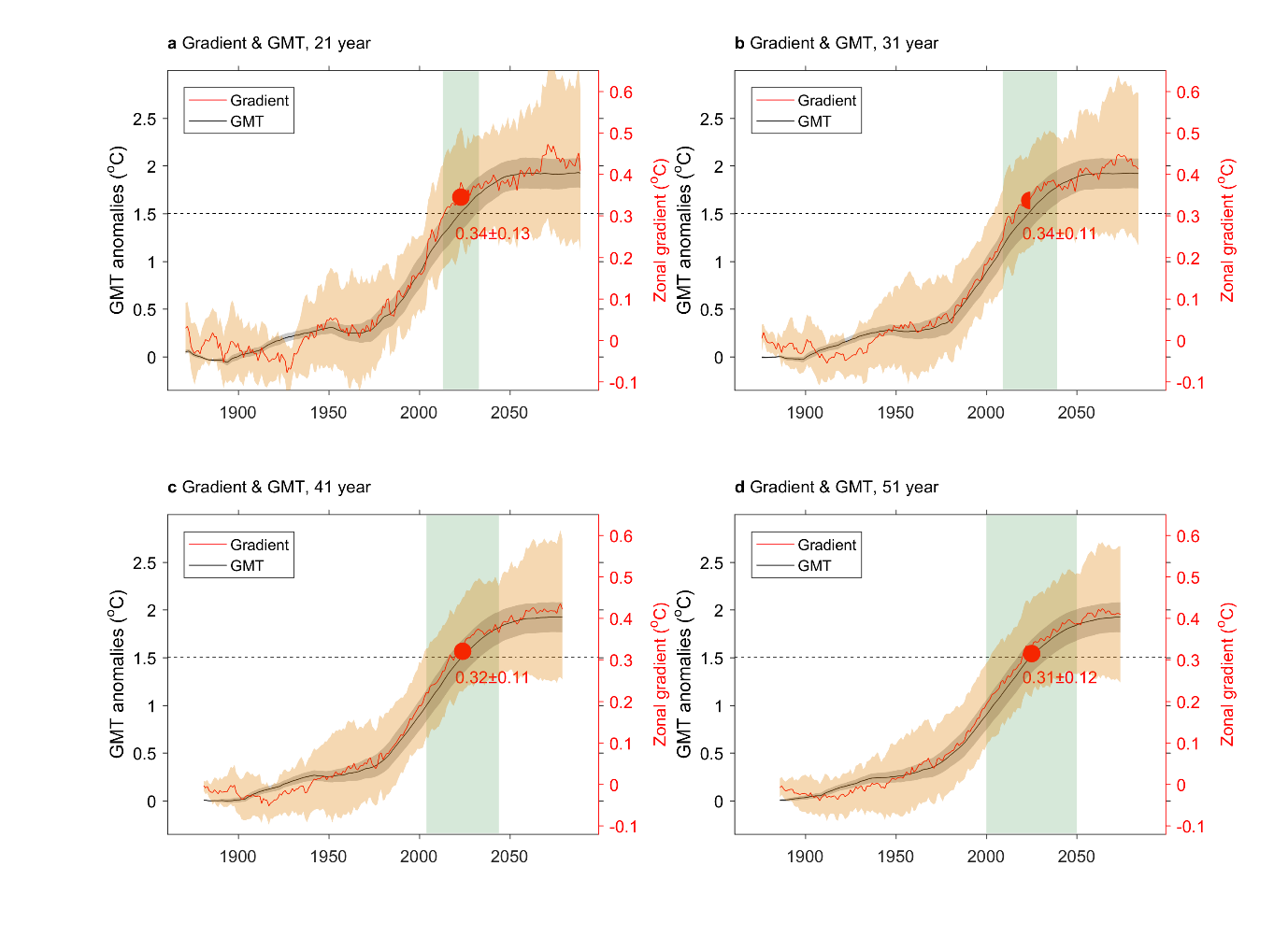


**Supplementary Figure 3 | Sensitivity of zonal temperature gradient to lengths of sliding windows.**  Evolution of multi-model ensemble averaged GMT anomalies (black curve), and the zonal temperature gradient (red curve), using 13 available models under the historical and RCP2.6 emission scenario. Shown are calculated using, **a-d,** 21-, 31-, 41-, and 51-year sliding windows for each model, respectively. The 90% confidence intervals for zonal temperature gradient (light red shades) and for GMT anomalies (grey shades) are based on Student’s *t*-distribution. The text label near the red filled circle indicates the averaged zonal temperature gradient centred at the 1.5 ^o^C warming (light shaded time window). The results show a very small difference using various window lengths.


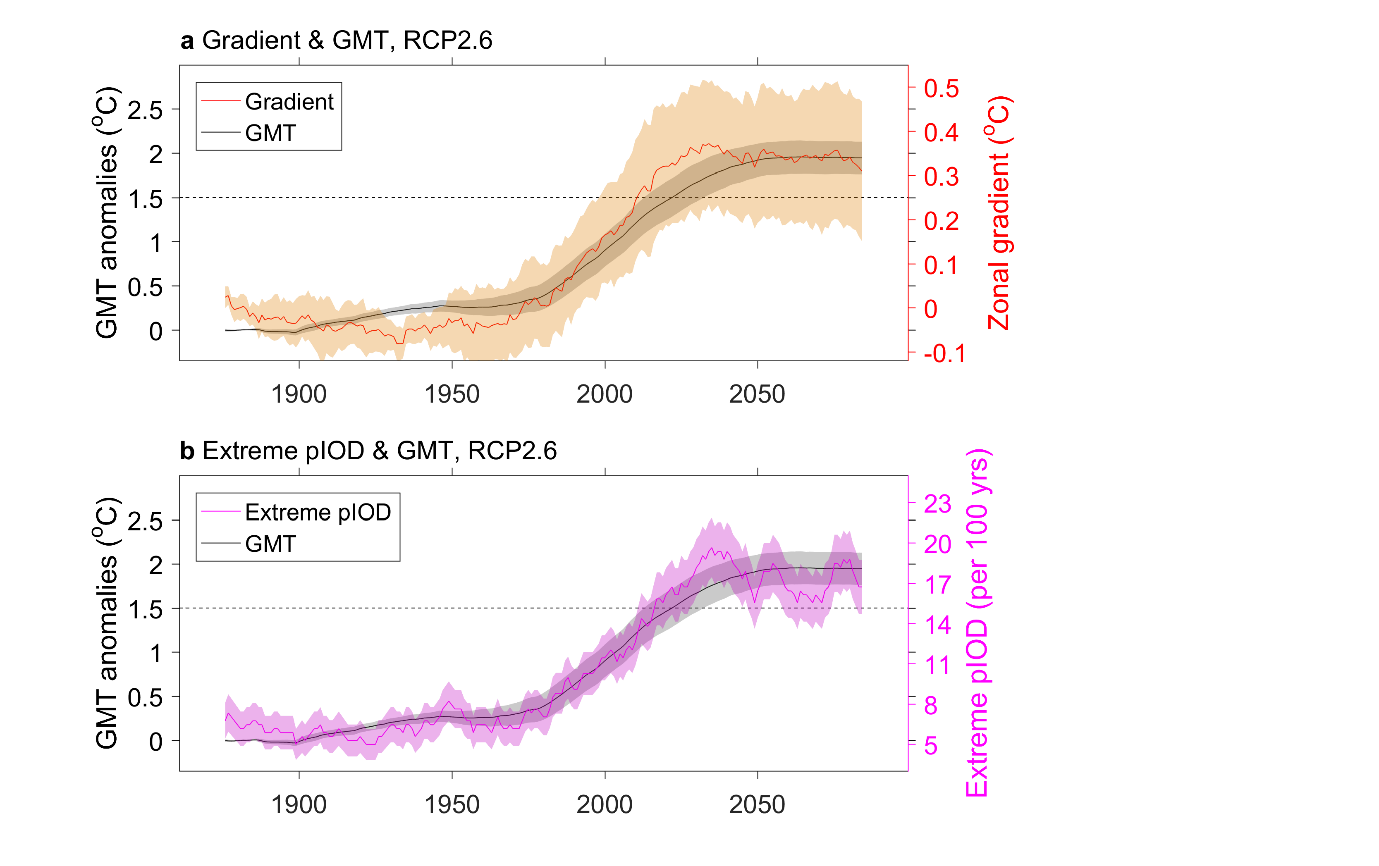


**Supplementary Figure 4 | Temporal evolution of multi-model ensemble mean changes under the RCP2.6 scenario. a**, GMT anomalies (black curve) and zonal temperature gradient anomalies at the equatorial Indian Ocean (red curve) referenced to the pre-industrial condition (1869–1899) and averaged over 31-year sliding windows from 1869–2099. Their 90% confidence intervals are indicated by grey and light red shades, respectively, based on a Student’s *t*-distribution. **b**, As **a**, but for the extreme pIOD frequency (purple curve, events per 100 years). The 90% confidence intervals (light purple shades) are estimated based on Poisson distribution (Methods). Results are based on the same models used in Fig.3 but without ‘CSIRO-Mk3-6-0’.


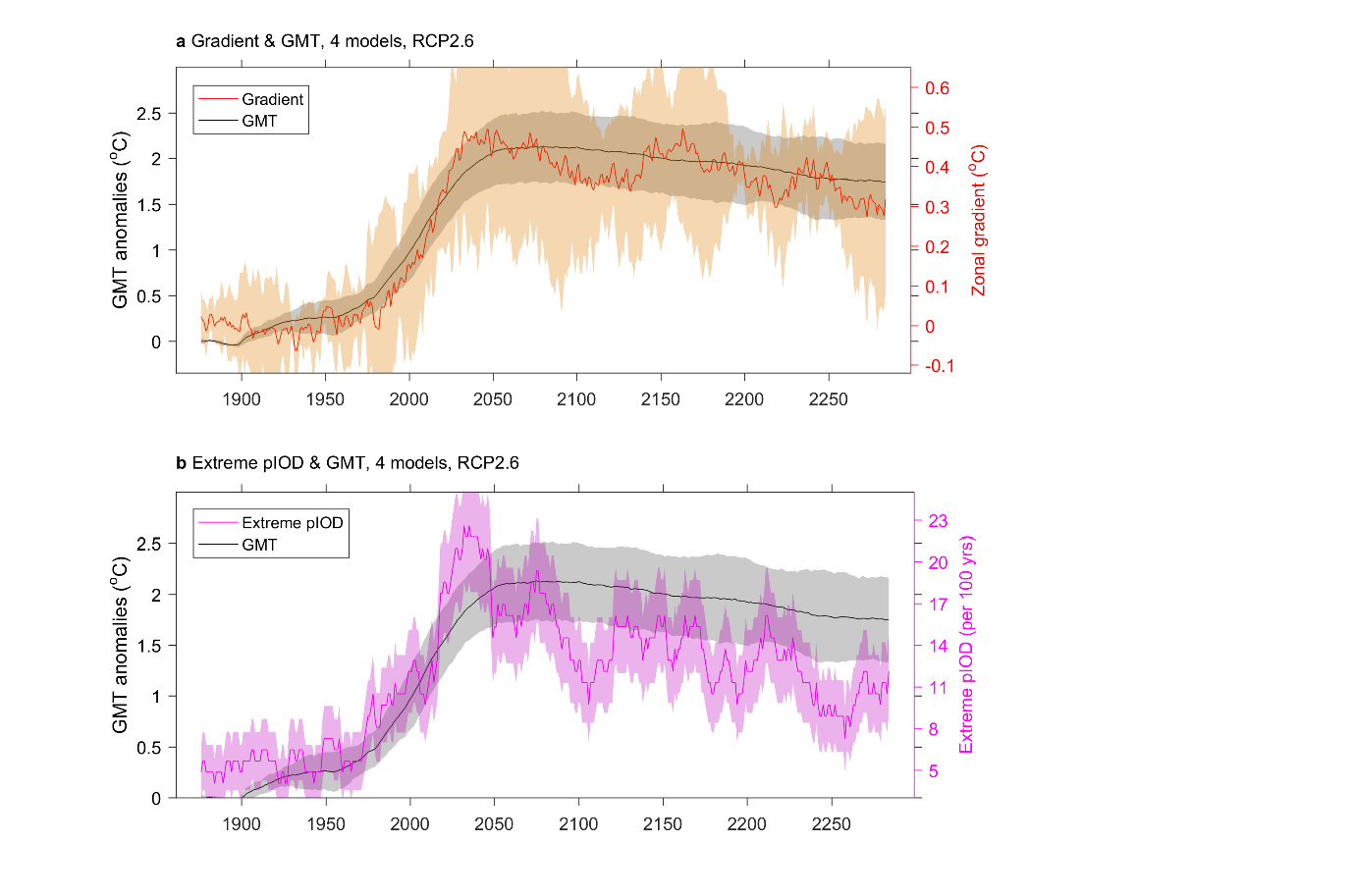


**Supplementary Figure 5 | Temporal evolution of multi-model ensemble mean changes under the RCP2.6 scenario**. **a,** GMT anomalies (black curve) and zonal temperature gradient anomalies **(**red curve) referenced to the pre-industrial condition (1869–1899) and averaged over 31-year sliding windows from 1869–2299. Their 90% confidence intervals are indicated by grey and light red shades, respectively, based on a Student’s *t*-distribution. **b**, The same as **a,** but for the extreme pIOD frequency (purple curve, events per 100 years). The 90% confidence intervals (light purple shades) are estimated based on Poisson distribution (Methods). Results are based on four available models that are run to year 2300.**Supplementary Table 1 | Comparison of the frequency in extreme pIOD events between pre-industry and 1.5 ^o^C warming world**.

|  | Extreme pIOD frequency  Pre-industrial period  Events per 100 years | Extreme pIOD frequency  1.5^o^C warming world  Events per 100 years |
| --- | --- | --- |
| bcc-csm1-1-m dt | 9.7 | 22.6 |
| CanESM2 τ_x_ dt | 0 | 12.9 |
| CESM1-CAM5 | 6.5 | 9.7 |
| CNRM-CM5 dt | 12.9 | 25.8 |
| CSIRO-Mk3-6-0 τ_x_ dt | 9.7 | 12.9 |
| FGOALS-s2 τ_x_ | 9.7 | 3.2 |
| GFDL-CM3 τ_x_ dt | 9.7 | 6.5 |
| HadGEM2-AO τ_x_ | 6.5 | 29.0 |
| IPSL-CM5A-LR τ_x_ dt | 6.5 | 6.5 |
| MIROC5 τ_x_ dt | 0 | 16.1 |
| MPI-ESM-LR τ_x_ dt | 3.2 | 9.7 |
| MPI-ESM-MR τ_x_ dt | 3.2 | 9.7 |
| NorESM1-ME dt | 6.5 | 9.7 |
| **Average** | 6.5 | 13.4 (106.2%) |

Models that can reach 1.5 ^o^C warming under RCP2.6 and are also able to generate extreme pIOD events are listed. Frequency is in the unit of events per 100 years. Models in red indicate a decrease in the frequency of extreme pIOD events in a 1.5 ^o^C warming world comparing to that in the pre-industrial period. Models with a “τ_x”_ indicate that zonal wind stress is available; models with a “dt” indicate that thermocline depth is available. The ensemble-mean frequency increase is statistically significant above the 95% confidence interval based on a Poisson distribution.

**Supplementary Table 2 | Comparison of the frequency in concurrent extreme events after 2050 in the 21^st^ century between RCP2.6 and RCP8.5**.

|  | Concurrent extremes  2050-2099, RCP2.6  Events per 100 years | Concurrent extremes  2050-2099, RCP8.5  Events per 100 years |
| --- | --- | --- |
| bcc-csm1-1-m | 4 | 4 |
| CanESM2 | 8 | 20 |
| CESM1-CAM5 | 12 | 22 |
| CNRM-CM5 | 10 | 24 |
| ~~CSIRO-Mk3-6-0~~ |  |  |
| FGOALS-s2 | 4 | 18 |
| GFDL-CM3 | 8 | 20 |
| ~~HadGEM2-AO~~ |  |  |
| ~~IPSL-CM5A-LR~~ |  |  |
| MIROC5 | 2 | 10 |
| ~~MPI-ESM-LR~~ |  |  |
| MPI-ESM-MR | 0 | 4 |
| NorESM1-ME | 10 | 4 |
| **Average** | 6.4 | 14.0 (118.8%) |

The concurrent extreme event is defined as an extreme pIOD event followed by an extreme El Niño event in the same year. Frequency is in the unit of events per 100 years. Models that crossed are not able to generate extreme El Niño event. Models in red indicate a decrease in the frequency of such events under RCP8.5 comparing to that under RCP2.6. The ensemble-mean frequency increase is statistically significant above the 95% confidence interval based on a Poisson distribution.
